# Methodology for Assessing the Probability of Corrosion in Concrete Structures on the Basis of Half-Cell Potential and Concrete Resistivity Measurements

**DOI:** 10.1155/2013/714501

**Published:** 2013-05-16

**Authors:** Lukasz Sadowski

**Affiliations:** Institute of Building Engineering, Wroclaw University of Technology, Plac Grunwaldzki 11, 50-377 Wroclaw, Poland

## Abstract

In recent years, the corrosion of steel reinforcement has become a major problem in the construction industry. Therefore, much attention has been given to developing methods of predicting the service life of reinforced concrete structures. The progress of corrosion cannot be visually assessed until a crack or a delamination appears. The corrosion process can be tracked using several electrochemical techniques. Most commonly the half-cell potential measurement technique is used for this purpose. However, it is generally accepted that it should be supplemented with other techniques. Hence, a methodology for assessing the probability of corrosion in concrete slabs by means of a combination of two methods, that is, the half-cell potential method and the concrete resistivity method, is proposed. An assessment of the probability of corrosion in reinforced concrete structures carried out using the proposed methodology is presented. 200 mm thick 750 mm  ×  750 mm reinforced concrete slab specimens were investigated. Potential *E*_corr_ and concrete resistivity *ρ* in each point of the applied grid were measured. The experimental results indicate that the proposed methodology can be successfully used to assess the probability of corrosion in concrete structures.

## 1. Introduction

Corrosion of the steel reinforcement in concrete is a crucial problem for the construction industry since it poses the most serious risk to the structural integrity of reinforced concrete structures. Inspection and monitoring techniques are needed to assess the corrosion of the reinforcement in order to maintain, protect, and repair buildings and bridge decks so that they remain safe [1, 2]. In the last few years much attention has been given to developing techniques for predicting the remaining service life of concrete structures [3]. Most of the reported research in this area focuses on the corrosion of concrete reinforcement [4].

**Figure 1:**
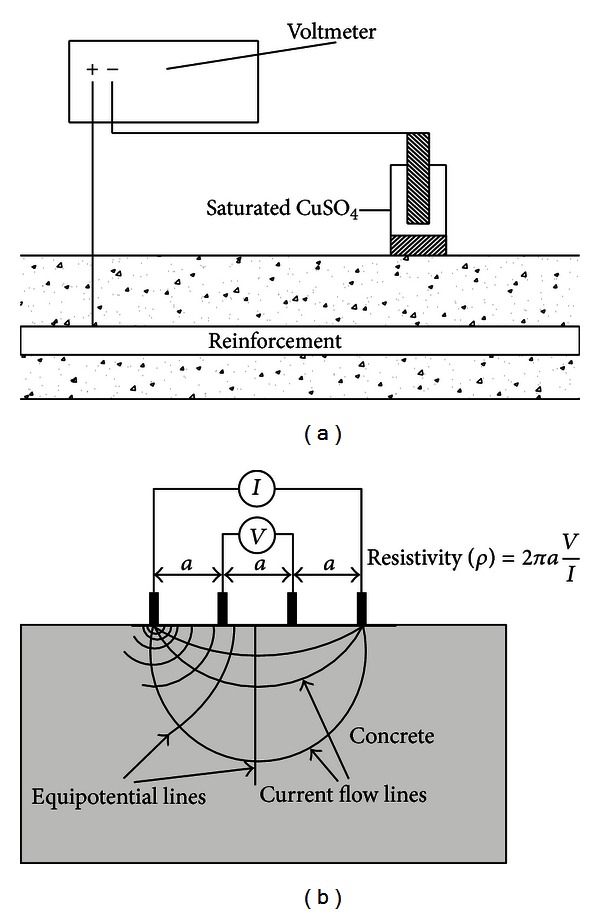
Existing corrosion methods: (a) half-cell potential measurement [27] and (b) concrete resistivity measurement [28].

Several electrochemical techniques for monitoring and assessing the corrosion of steel in concrete structures were presented in [5–7]. The most popular method of *in situ* corrosion testing is the half-cell potential measurement, the idea of which is illustrated in Figure 1(a). The use of this method and the interpretation of its results are described in ASTM C876 [8]. Such corrosion potential measurements, however, should be supplemented with other nondestructive testing methods [9–12]. The use of half-cell potential measurements for determining the probability of corrosion in concrete was extensively described by Flis et al. [13], Grantham et al. [14], and Žvica [15]. The latter also presented dependences between the rate of reinforcement corrosion and temperature. The effectiveness of the test was studied in [16]. It should be noted that half-cell potential values merely provide information about the probability of corrosion and not about the rate of corrosion.

It is well known that the probability of corrosion in concrete structures depends on the ionic conductivity of the concrete electrolyte, the humidity, the temperature, and the quality of the concrete cover. The ionic conductivity is measured quantitatively as the resistivity of the concrete [17]. Concrete resistivity **ρ** ranges widely from 10^1^ to 10^6^ Ω m, depending on mainly the moisture content [18] and the material of the concrete [19, 20]. One of the promising techniques of measuring concrete resistivity is shown in Figure 1(b). As shown by Feliu et al. [21], concrete resistivity **ρ** is inversely proportional to the corrosion rate. This was confirmed by Glass et al. [22] who showed that the effect of mortar resistivity is strongly dependent on the relative humidity of the environment, while López et al. [23] showed that the amount of pores in concrete determines its resistivity **ρ** and corrosion rate. The four-point resistivity method enables one to determine the severity of corrosion in a quick and nondestructive manner. Morris et al. [24] found that rebars undergo active corrosion when concrete resistivity **ρ** is below 10 kΩcm, whereas at concrete resistivity **ρ** above 30 kΩcm the probability of their corrosion is low. Extensive research on the resistivity technique, covering experimental analyses [25] and an analysis of the effects of geometry and material properties [26], was done by Zhang et al.

It should be noted that in the literature there are only a few papers in which the probability of corrosion is determined using both concrete resistivity measurements and half-cell potential mapping. One of them is the paper by Millard and Sadowski [29] in which in order to determine the degree of corrosion of the reinforcement, the resistivity of the concrete is measured by the electrodes used in the half-cell potential method. The combined use of half-cell potential and resistivity measurements was presented in [27]. In [28] it was shown that the combination of the method described in [29] and the conventional method of measuring concrete resistivity could be a reliable tool for directly determining the corrosion rate of the reinforcement in concrete. In paper [30] for this purpose Vedalakshmi et al. used the galvanostatic pulse technique with electrochemical impedance spectroscopy and the weight-loss method. Rhazi [31] measured concrete resistivity **ρ** in the same locations where half-cell potentials were measured, but it should be noted that the measurements were carried out on a concrete bridge deck covered with asphalt.

Considering that in the literature it is hard to find a systematic methodology for assessing the probability of corrosion in concrete slabs through combined half-cell potential and concrete resistivity measurements, this paper proposes such a methodology based on the combined use of the four-point Wenner concrete resistivity method and the half-cell potential method.

## 2. Methodology for Assessing Probability of Corrosion in Concrete Slabs

**Figure 2:**
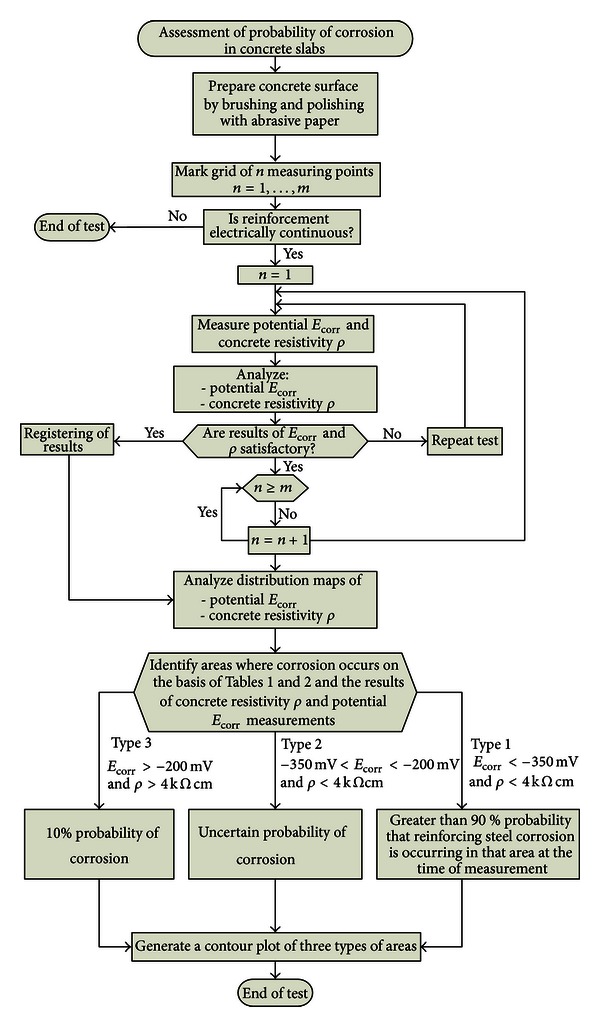
Assessment of probability of corrosion in concrete slabs through half-cell potential and concrete resistivity measurements.

The proposed methodology for assessing the probability of corrosion in concrete slabs through a combination of two methods, that is, the half-cell potential method and the concrete resistivity method, is illustrated graphically in Figure 2. 

Before measurements, the surface of the concrete slab should be prepared by brushing and polishing with abrasive paper, and a grid of *n* measuring points spaced at every 75 mm should be marked on the slab surface. Then the electrical continuity of the reinforcement is checked in three randomly selected grid points. Subsequently potential *E*_corr_ and concrete resistivity **ρ** are measured. If the results of the *E*_corr_ and **ρ** measurements are satisfactory, they are processed using the specialized software and maps of the distribution of the parameter values on the slab surface are produced. The distribution maps of potential *E*_corr_ and concrete resistivity **ρ** should be examined. Table 2 summarizes the typical interpretation of half-cell potential readings [8]. The dependences between reinforcement corrosion probability and concrete resistivity measured by the four-point Wenner method are shown in accordance with [32] in Table 3. If there is a probability of corrosion, one should identify the areas in which corrosion may occur. On the basis of Tables 1 and 2 and the results of the concrete resistivity **ρ** and potential *E*_corr_ measurements, three areas of different types can be generated:

- area type 1—low values of both parameters, greater than 90% probability that reinforcing steel corrosion is occurring in that area at the time of measurement;
- area type 2—low values of concrete resistivity **ρ** and high values of measured potential *E*_corr_, an uncertain probability of corrosion;
- area type 3—high values of both parameters, a 10% probability of corrosion.

Finally, a contour plot of three types of areas (type 1, type 2, and type 3) should be generated.

The corrosion probability assessment can be practically verified through test pits made in selected places and visual inspection [33]. 

## 3. Exemplary Application of the Proposed Methodology

### 3.1. Materials and Methods

**Figure 3:**
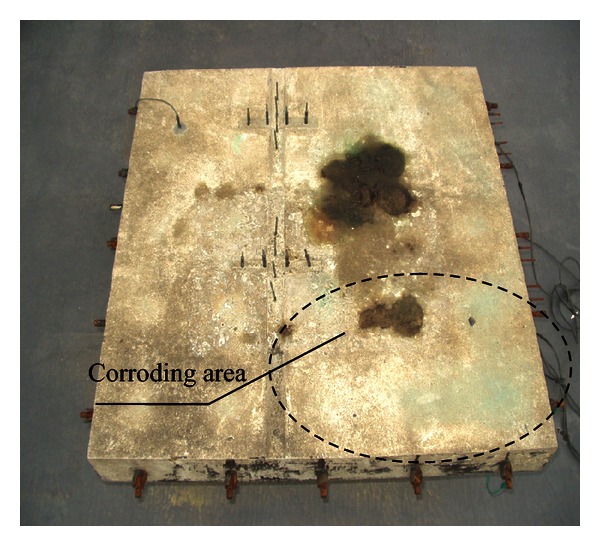
General view of concrete slab specimen.

An exemplary application of the proposed methodology is presented below. 200 mm thick 750 mm × 750 mm concrete slab specimen was investigated (Figure 3). The concrete had been designed to strengthen class C 20/25. Portland cement CEM I 42.5R, well-graded sand, and crushed blue granite with a maximum total grading of 5 mm, consistency S3, and w/c = 0.5 had been used to cast the slab specimens. A reinforcement mesh made of A-III 34GS steel rebars 10 mm in diameter spaced at every 150 mm with a 50 mm cover had been embedded inside each slab. The concrete slab specimen was placed in a normal atmosphere and was subjected to a two-hour spray wetting with a sodium chloride solution cycle followed by twenty hours drying cycle to generate corroding area presented in Figure 3. 

Measurements were carried out after 90 days of concrete curing, except for compressive strength tests which were done after 28 days. The concrete slabs cured at an air temperature of +18°C (±3°C) and a relative air humidity of 60%. It is important to measure concrete resistivity in constant temperature-humidity conditions since, as shown in [34], with each degree Celsius relative humidity increases by 3% [35]. After the concrete slabs were labelled, a 750 mm × 750 mm test area was demarcated on each of them and a grid of points spaced at every 75 mm was marked on each of the slabs. The columns were denoted with letters from A to I, and the rows were numbered from 1 to 9. A total of 81 measuring points were marked on the surface.

**Figure 4:**
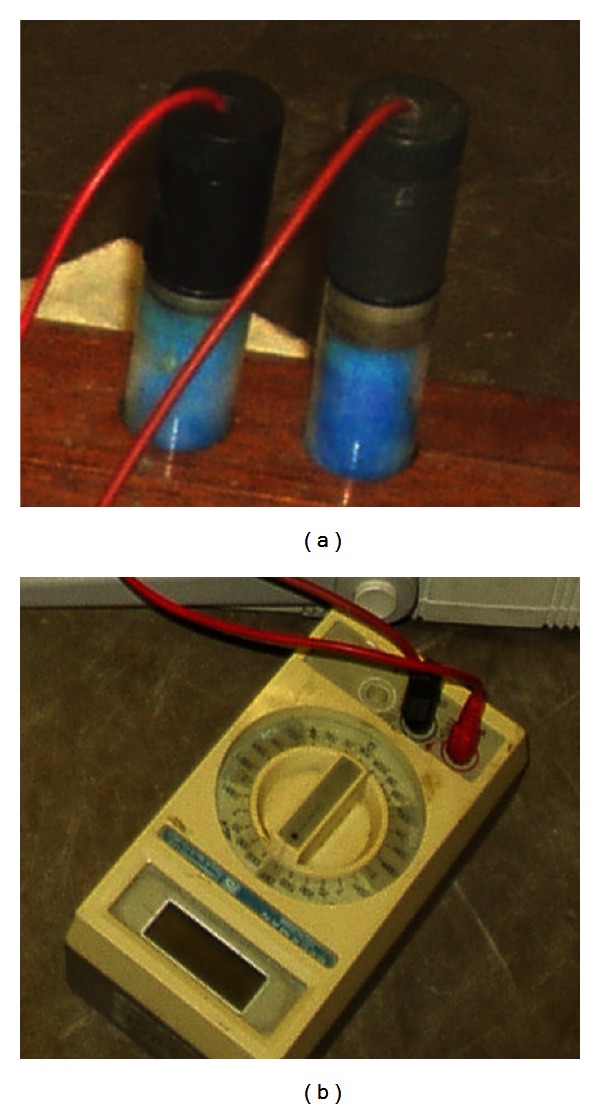
Half-cell potential measurements: (a) copper/copper sulphate electrode and (b) digital voltmeter.

Prior to half-cell potential measurements, the electrical continuity of the reinforcement was checked at three randomly selected grid points. The potential differences in the three points were measured with a digital voltmeter, and all the measured values were found to be below 1 mV. The copper/copper sulphate electrode used in this test is shown in Figure 4(a), and the digital voltmeter with high input impedance is shown in Figure 4(b). Before the measurements, the concrete surface was prepared by brushing and polishing with abrasive paper. The area where each measurement was to be taken was wetted with tap water to ensure better electrical contact. Then the reference electrode and the reinforcement mesh were connected to the high-impedance voltmeter, and the reference electrode was placed on the surface of the concrete.

**Figure 5:**
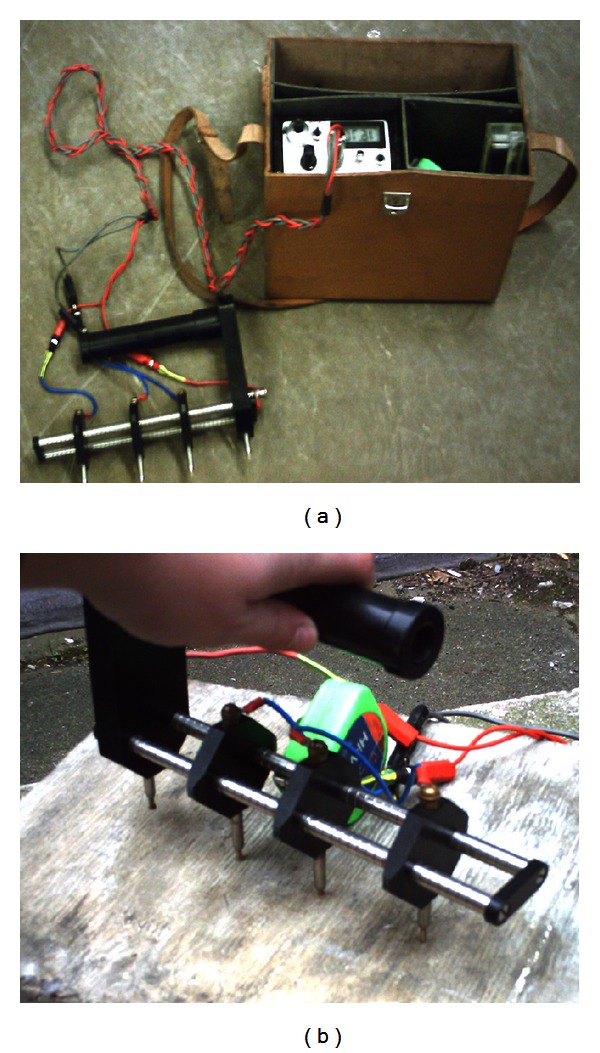
Concrete resistivity measurement: (a) test setup and (b) measurement.

The concrete resistivity measurements were carried out at frequencies in a range of 50–1000 Hz. Before the measurements, the equipment was calibrated using a 1 kΩ calibration bar. A single deviation was not larger than 2% and the average deviation amounted to less than 1%. Concrete resistivity *ρ* was measured in each point of the grid (Figure 5).

### 3.2. Results 

#### 3.2.1. Half-Cell Potential Measurements

**Figure 6:**
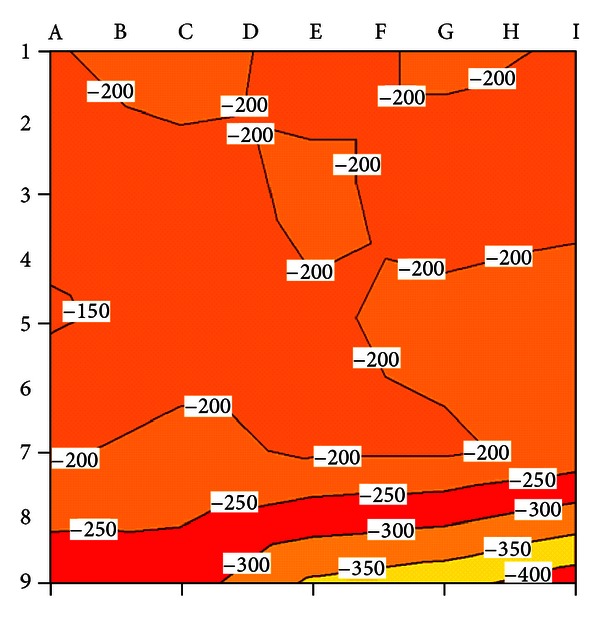
Contour plot of potential *E*_corr_.

Exemplary results of the half-cell potential measurements are shown in Table 3. The results of the half-cell potential measurements were plotted on a contour map for visual interpretation (Figure 6). It is evident that potential *E*_corr_ is low (<−350 mV) in the area around measuring points 7 to 9 from E to I, which indicates a 95% probability of corrosion. In the other measuring points, potential *E*_corr_ is high (−350 mV ≤ *E*_corr_ ≤ −200 mV), which indicates a 10% or uncertain probability of corrosion.

#### 3.2.2. Concrete Resistivity Measurements

**Figure 7:**
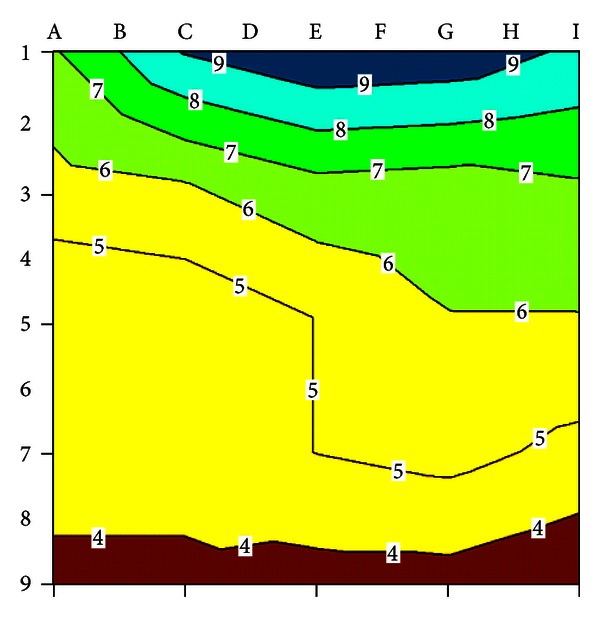
Contour plot of concrete resistivity *ρ*.

The results of the concrete resistivity measurements are shown in Table 4. The results of the concrete resistivity measurements were plotted on an equipotential contour map for visual interpretation (Figure 7). It is evident that concrete resistivity **ρ** is low (<5 kΩcm) in the area around measuring points 5 to 9 from A to I, which indicates a very high probability of corrosion. In the other measuring points, concrete resistivity **ρ** is high (>5 kΩcm), indicating a high or moderate probability of corrosion. 

### 3.3. Statistical Analyses of Test Results

**Figure 8:**
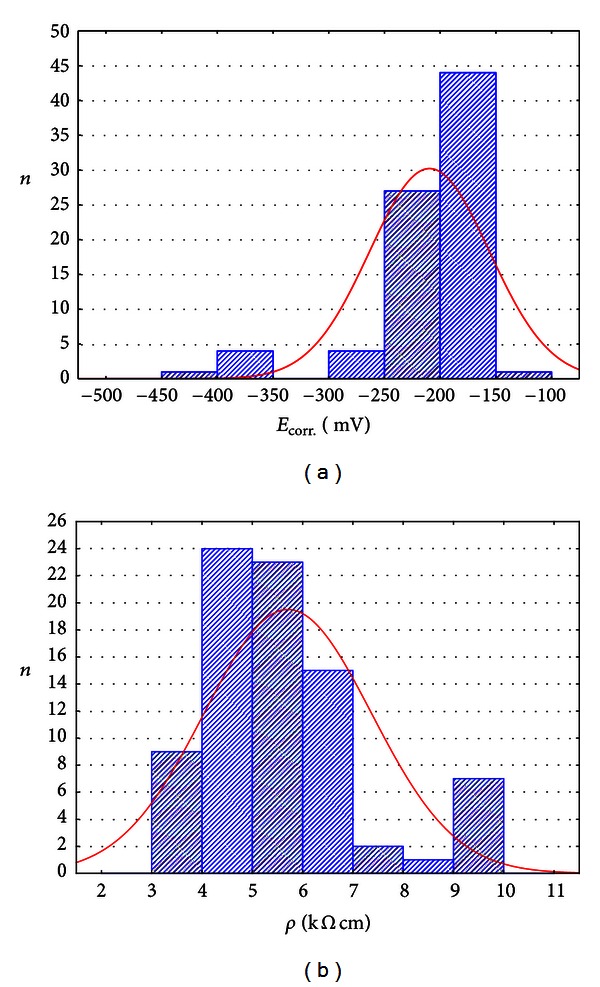
Histogram of (a) potential *E*_corr_ and (b) concrete resistivity **ρ**.

Selected statistical characteristics of the parameters determined by half-cell potential and concrete resistivity measurements are shown in Table 5. As it appears from the histograms presented in Figure 8 and statistical characteristics shown in Table 5, the half-cell potential method yielded potential *E*_corr_ ranging from −432 to −144 mV. The average value of potential *E*_corr_ was −211.09 mV with standard deviation of 53.46 mV. 

Concrete resistivity *ρ* determined by concrete resistivity measurements ranged from 3.14 to 9.99 kΩcm. The average value of concrete resistivity *ρ* was 5.68 kΩcm with standard deviation of 1.66 kΩcm. 

### 3.4. Discussion

**Figure 9:**
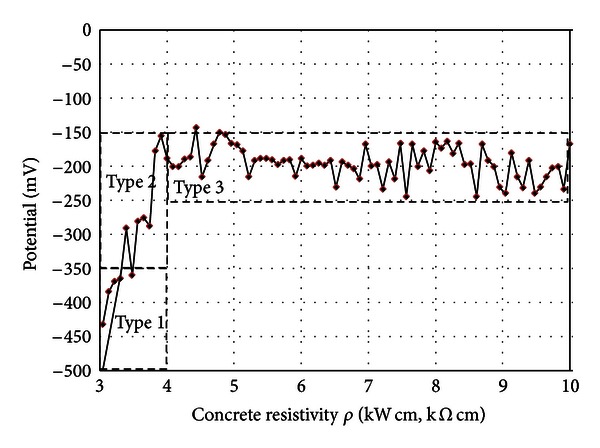
Concrete resistivity **ρ** versus potential *E*_corr_.

The dependence between concrete resistivity **ρ** and potential *E*_corr_, measured on the concrete slab surface, is shown in Figure 9. One should note that potential *E*_corr_ sharply increases for resistivity **ρ** below 4 kΩcm while above 4 kΩcm, it oscillates between −150 and −250 mV.

**Figure 10:**
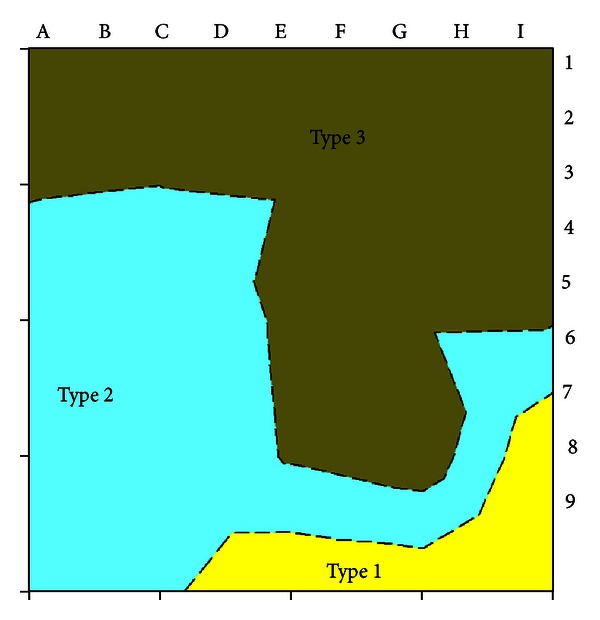
A contour plot of three areas of different types.

On the basis of Tables 1 and 2 and the results of the concrete resistivity **ρ** and potential *E*_corr_ measurements, three areas of different types were generated (Table 6) as it has been presented in Section 2. A contourplot of the three areas is shown in Figure 10. The area around measuring points 6 to 9 from C to I is of type 1, which means that there is a 90% probability of corrosion. In the measuring points, potential *E*_corr_ is below −250 mV and concrete resistivity **ρ** is above 4 kΩcm. The area around measuring points 3 to 9 from A to E is of type 2, which means that there is a uncertain probability of corrosion. In the measuring points, potential *E*_corr_ is between −150 mV and −350 mV and concrete resistivity **ρ** is above 4 kΩcm. The area around measuring points 1 to 7 from A to I is of type 3, which means that there is an 10% probability of corrosion. In the measuring points, potential *E*_corr_ is above −250 mV and concrete resistivity **ρ** is above 5 kΩcm.

## 4. Conclusion

A methodology for assessing the probability of corrosion in concrete slabs based on a combination of two nondestructive methods, that is, the half-cell potential method and the concrete resistivity method, was briefly described. Comparative tests were carried out using the two methods to determine the probability of corrosion in model test concrete slab specimens. The experimental results showed that the two nondestructive techniques can be used together in order to obtain maximum information about the probability of corrosion in a tested structure.

This study was motivated by the engineer's need for a combination of the half-cell potential mapping technique and concrete resistivity measurements to more accurately assess the probability of corrosion. The combined techniques can be used in both the field and the laboratory environment. Moreover, they can be automated and integrated into monitoring systems for new or existing reinforced concrete structures. However, it is still recommended to perform additional tests for other rebar diameters, different aggregate grading, and a wider range of covers.

## Figures and Tables

**Table 1:** Dependence between potential and corrosion probability [8].

Potential *E*_corr_	Probability of corrosion
*E*_corr_< −350 mV	Greater than 90% probability that reinforcing steel corrosion is occurring in that area at the time of measurement

−350 mV ≤*E*_corr_≤ −200 mV	Corrosion activity of the reinforcing steel in that area is uncertain

*E*_corr_>−200 mV	90% probability that no reinforcing steel corrosion is occurring in that area at the time of measurement (10% risk of corrosion)

**Table 2:** Dependence between concrete resistivity and corrosion probability [34].

Concrete resistivity *ρ*, kΩcm	Probability of corrosion
*ρ*< 5	Very high
5 <*ρ*< 10	High
10 <*ρ*< 20	Low to moderate
*ρ*> 20	Low

**Table 3:** Potential *E*_corr_.

Nr	Potential *E*_corr_, mV								
	A	B	C	D	E	F	G	H	I
1	−192	−168	−154	−151	−144	−156	−178	−192	−288
2	−240	−167	−169	−167	−168	−187	−201	−216	−276
3	−234	−168	−198	−189	−178	−189	−200	−215	−281
4	−201	−178	−201	−190	−189	−191	−200	−199	−291
5	−168	−165	−181	−191	−192	−189	−194	−192	−360
6	−231	−174	−182	−204	−201	−199	−194	−198	−365
7	−203	−164	−198	−219	−232	−231	−199	−196	−369
8	−216	−167	−197	−207	−216	−245	−234	−192	−384
9	−192	−168	−201	−231	−240	−245	−219	−216	−432

**Table 4:** Concrete resistivity *ρ*.

Nr	Concrete resistivity *ρ*, kΩcm								
	A	B	C	D	E	F	G	H	I
1	6.91	5.62	4.56	4.56	4.34	4.12	4.08	4.39	3.77
2	9.11	5.96	4.63	4.57	4.56	4.34	4.23	4.38	3.76
3	9.89	5.98	4.85	4.78	4.67	4.22	5.03	5.02	3.75
4	9.88	5.99	5.99	4.23	4.78	4.89	5.65	5.33	3.71
5	9.99	6.02	6.98	4.8	4.76	5.02	5.43	5.35	3.74
6	9.45	6.25	6.71	5.43	4.23	5.03	5.68	5.67	3.67
7	9.77	6.66	6.77	5.55	7.51	5.42	5.43	5.21	3.62
8	9.73	6.71	6.88	6.01	7.01	5.98	5.67	4.88	3.61
9	8.79	6.91	6.93	6.96	6.98	6.91	5.96	4.71	3.14

**Table 5:** Selected statistical characteristics of parameters determined by half-cell potential and concrete resistivity measurements.

	Statistical characteristics			
	Average	Standard deviation	Minimum	Maximum
Potential *E*_corr_, mV	−211.09	53.46	−432	−144
Concrete resistivity *ρ*, kΩcm	5.68	1.66	3.14	9.99

**Table 6:** Proposed types of corrosion probability.

	Potential *E*_corr_, mV			
		*E*_corr_<−350	− 350≤*E*_corr_≤ − 200	−200≤*E*_corr_
Concrete resistivity *ρ*, kΩcm	*ρ*< 4	Type 1	Type 2	Type 2
	4 <*ρ*< 5			Type 3
	*ρ*> 5			Type 3
